# Umbilical Cord Mesenchymal Stem Cell-Derived Extracellular Vesicles Attenuate Oxidative Damage via the miR-191-5p/DAPK1/AKT Axis in Type 2 Diabetes

**DOI:** 10.34133/bmr.0224

**Published:** 2025-07-03

**Authors:** Anran Li, Cong Chen, Tongjia Zhang, Yuxin Tian, Yifan Cao, Xiaoming Zhao, Liping Wang

**Affiliations:** ^1^ National Engineering Laboratory for AIDS Vaccine, Key Laboratory for Molecular Enzymology and Engineering, the Ministry of Education, School of Life Sciences, Jilin University, Changchun 130012, China.; ^2^ Scientific Research Center, China-Japan Union Hospital of Jilin University, Changchun 130012, China.

## Abstract

Human umbilical cord mesenchymal stem cell extracellular vesicles (hucMSC-EVs) exhibit remarkable potential for alleviating type 2 diabetes mellitus (T2DM). However, the role of hucMSC-EVs in T2DM, particularly concerning oxidative damage to pancreatic β cells, remains underexplored. This study utilized a high-fat diet and streptozotocin (STZ)-induced T2DM mouse model and an STZ-induced INS-1 cell damage model to investigate the effects and mechanisms of hucMSC-EVs. In the T2DM mouse model, hucMSC-EVs effectively lowered blood glucose levels, improved lipid metabolism disorders, and preserved liver function. Moreover, hucMSC-EVs enhanced insulin sensitivity and mitigated oxidative damage. Histological analysis confirmed that hucMSC-EVs marked alleviated liver, kidney, and pancreatic tissue damage. In vitro studies demonstrate that hucMSC-EVs enhance glucose absorption and glycogen synthesis in an insulin-resistant HepG2 model and stimulated insulin secretion in INS-1 cells under high-glucose conditions. In the STZ-induced INS-1 oxidative damage model, hucMSC-EVs protect against oxidative damage by increasing antioxidant enzyme activities, reducing reactive oxygen species production, and decreasing cell apoptosis. The effects were partially mediated by the activation of the phosphatidylinositol 3-kinase (PI3K)/AKT and signal transducer and activator of transcription (STAT) signaling pathways, as well as the up-regulation of key antioxidant proteins such as Nrf2, SOD1, and Bcl2. Further research revealed that miR-191-5p, which is enriched in hucMSC-EVs, targets DAPK1 to activate the PI3K/AKT pathway, thereby contributing to the protective effects against oxidative damage. These findings highlight the critical role and underlying mechanisms of hucMSC-EVs in ameliorating metabolic dysfunction in T2DM, particularly the protective effects against oxidative damage, thus providing a novel strategy for the treatment of T2DM.

## Introduction

Type 2 diabetes mellitus (T2DM) is the most common type of diabetes [1], characterized by insulin resistance (IR) and aberrant pancreatic β cell function. Dyslipidemia, hyperglycemia, and other metabolic dysregulation contribute to IR and/or pancreatic β cell dysfunction through pathways associated with inflammation, endoplasmic reticulum (ER) stress, oxidative damage, and ectopic lipid deposition [2,3]. As metabolic abnormalities affect multiple organs, the majority of patients with T2DM develop at least one complication during their lifetime, such as diabetic retinopathy, nephropathy, or neuropathy [4]. Currently, common medications for treating T2DM include thiazolidinediones, metformin, α-glucosidase inhibitors, sulfonylurea derivatives, exogenous insulin, and glucagon-like peptide-1 receptor agonists. Although these drugs can lower blood glucose levels and alleviate metabolic syndrome by improving impaired glucose tolerance, existing treatments can only temporarily control blood glucose levels and are often accompanied by adverse effects. For example, thiazolidinedione drugs may interfere with adipocyte differentiation and triglyceride (TG) storage, leading to weight gain [5]. Although metformin effectively reduces blood glucose levels, its efficacy in improving inflammatory markers, IR, and vascular function is relatively limited. Moreover, sulfonylurea-derived drugs carry a risk of causing severe hypoglycemia [6]. Additionally, long-term use of exogenous insulin may further impair β cell function, exacerbate IR, and prevent the effective control of diabetic complications.

Under normal physiological conditions, the intracellular antioxidant system effectively neutralizes reactive oxygen species (ROS), thereby maintaining redox balance [7,8]. However, when ROS production exceeds the clearance capacity of the cellular antioxidant system, oxidative damage occurs, potentially triggering various pathophysiological states including T2DM [9]. Chronic hyperglycemia in β cells disrupts normal physiological glucose metabolism through oxidative phosphorylation, leading to mitochondrial dysfunction and increased ROS production, which ultimately results in β cell failure [10,11]. Oxidative damage can inhibit the activity of the phosphatidylinositol 3-kinase (PI3K)/AKT pathway, weakening survival signals and further exacerbating β cell damage [12,13]. PI3K/AKT pathway inhibition also affects insulin receptor phosphorylation, reduces insulin signal transduction, and leads to IR. Furthermore, oxidative damage can influence Janus kinase (JAK)/signal transducer and activator of transcription (STAT) pathway activity. The JAK/STAT pathway, particularly STAT3 and STAT5, is crucial for maintaining β cell function and regulating insulin signal transduction. Abnormalities in the STAT pathway can further aggravate β cell damage and promote β cell failure [14,15]. Oxidative damage not only affects β cell function but also induces IR in peripheral tissues through multiple mechanisms [16,17]. For instance, oxidative damage can activate protein kinase C, leading to the phosphorylation of insulin receptor substrates (IRSs), thereby inhibiting insulin signal transduction. Concurrently, oxidative damage generates advanced glycation end products, which, upon binding to their receptor (RAGE), activate multiple signaling pathways, including the mitogen-activated protein kinase (MAPK), extracellular signal-regulated kinase (ERK), and PI3K signaling pathways, further exacerbating IR. Nuclear factor E2-related factor 2 (Nrf2) and its downstream molecule heme oxygenase-1 (HO-1) constitute a critical antioxidant defense system of cells. In patients with T2DM, inhibition of the Nrf2/HO-1 axis reduces ROS clearance, exacerbates oxidative damage, induces ER stress, and results in cellular damage and apoptosis [18].

The potential of mesenchymal stem cells (MSCs) for the treatment of T2DM has been demonstrated and is related to their multilineage differentiation, immunomodulation, and anti-inflammatory effects [19,20]. However, issues such as low survival and implantation efficiency, as well as difficulties in differentiation and functional maintenance remain unresolved [21,22]. Ongoing research has revealed that the functional activity of MSCs occurs primarily via the paracrine pathway, and extracellular vesicles play a critical role in this process. Human umbilical cord MSC extracellular vesicles (hucMSC-EVs) have shown potential in regulating insulin secretion, protecting pancreatic β cells, and enhancing insulin sensitivity. However, the intricate mechanisms underlying these effects remain only partially elucidated, limiting their application in clinical settings. Extracellular vesicles are characterized by their small vesicle structure and ability to carry various biomolecules that play crucial roles in intercellular communication. These nano-sized vesicles not only convey biological information to recipient cells, regulating their gene expression and metabolic state, but also exert precise regulation over multiple cellular processes through small RNA molecules, such as microRNAs (miRNAs). miRNAs are endogenous small RNAs approximately 20 to 24 nucleotides in length that play significant roles in cellular metabolism, proliferation, and apoptosis by regulating the expression of target mRNAs [23,24]. For example, extracellular vesicles enriched with miR-1249-3p from natural killer cells alleviate IR and inflammation in vivo and in vitro by inhibiting SKOR1 expression [25]. Similarly, M2-polarized macrophage-derived extracellular vesicles containing miR-690 improve insulin sensitivity in obese mice [26].

In this study, we investigated the feasibility and effectiveness of hucMSC-EVs in alleviating T2DM. Our findings demonstrated that hucMSC-EVs significantly mitigates IR and oxidative damage in T2DM mouse models, as well as in STZ-induced oxidative damage model in the INS-1 cells. HucMSC-EVs activate the PI3K/AKT and STAT signaling pathways and improve ROS elevation and cell apoptosis caused by oxidative damage. Moreover, miR-191-5p, a highly abundant miRNA in hucMSC-EVs, plays a crucial role in combatting oxidative damage. Our results shed light on the function and mechanism of hucMSC-EVs in reducing oxidative damage in pancreatic β cells, providing a theoretical foundation for the therapeutic application of hucMSC-EVs in the treatment of T2DM.

## Materials and Methods

### Cell culture

Human hepatoma HepG2, rat insulinoma INS-1, and 293T cells were purchased from the Cell Bank of the Chinese Academy of Science and cultured in Dulbecco’s modified Eagle’s medium (DMEM) (MeilunBio, Dalian, China) or RPMI 1640 (MeilunBio) containing 10% fetal bovine serum (FBS) (MeilunBio). hucMSCs were cultured in MEM (Meilun Bio) supplemented with 10% FBS (Hyclone, CAS: SH30406.05, Los Angeles, CA, USA) from which the extracellular vesicles had been removed by ultracentrifugation. All cell lines were maintained in a 37 °C humidified incubator with 5% CO_2_. HepG2 cells were incubated with glucosamine hydrochloride (18 mM; Aladdin, Shanghai, China) for 24 h to establish an insulin-resistant cell model. INS-1 cells were incubated with streptozotocin (STZ; Macklin, Shanghai, China) at 15.15 mM for the oxidative stress cell model.

### Exosome purification and characterization

Select hucMSCs (passages 3 to 8) were cultured, and the cells were closely monitored to ensure that they consistently retain a long spindle-like shape, adhered firmly to the surface, and presented no abnormal manifestations such as vacuoles. When the cells reached 70% to 80% confluency, the original culture medium was discarded and the cells were washed 3 times with phosphate-buffered saline (PBS). The cells were cultured for an additional 48 h in serum-free MEM. Subsequently, culture medium was collected and centrifuged at 2,000*g* for 30 min to remove cell debris, and then centrifuged at 10,000*g* for 45 min to eliminate larger vesicles. The supernatant was filtered through a 0.22-μm filter (Biofil, Guangzhou, China). Ultracentrifugation was performed at 100,000*g* for 90 min to collect the hucMSC-EVs. All ultracentrifugation experiments were conducted using an Optima L-80XP system (Beckman Coulter, Florida, USA). The protein concentration in hucMSC-EVs was quantified using a bicinchoninic acid (BCA) assay. For the nanoparticle tracking analysis, the size and number of hucMSC-EVs were detected using flow systems (Apogee, Northwood, UK). Morphological observations were performed using a Hitachi HT7820 microscope (Hitachi, Tokyo, Japan). The exosomal markers TSG101, CD9, and CD81 were determined via Western blotting. To determine whether hucMSC-EVs could be internalized by cells, INS-1 and HepG2 cells were treated with hucMSC-EVs and stained with the red fluorescent cell membrane dye, Dil (Beyotime, Jiangsu, China).

### Induction of the T2DM animal model

All experimental protocols were approved by the Animal Ethics Committee of the School of Life Sciences at Jilin University (YNPZSY2024040). Six-week-old male BALB/c mice (20 to 25 g) were purchased from Liaoning Changsheng Biotechnology Co. Ltd. The mice were housed under a 12-h light/dark cycle at an ambient temperature of 22 to 25 °C. After 1 week of acclimatization, the mice were divided into 2 feeding groups: The control group continued to receive a normal diet, while the remaining mice were fed a 60% high-fat diet (HFD). After 26 d of group feeding, HFD-fed mice were fasted for 12 h (water was not restricted). Subsequently, HFD-fed mice received daily intraperitoneal injections of STZ at a dose of 40 mg/kg (dissolved in 0.1 M citrate buffer, pH 4.5) for 3 consecutive days to induce T2DM. Fasting blood glucose (FBG) levels were measured on the fourth day after the last STZ injection (i.e., on day 32 of group feeding). Mice were fasted for 12 h, and then FBG levels were determined using a glucometer (Yuyue, Jiangsu, China). Mice with FBG levels exceeding 11.0 mM were classified as having T2DM. To further validate the T2DM model, the oral glucose tolerance (OGTT) and insulin tolerance (ITT) tests were conducted. For the OGTT, the mice were fasted for 16 h and then orally administered 1 g/kg glucose via gastric gavage. Blood glucose levels were measured at 0, 30, 60, 90, and 120 min. For the ITT, mice were fasted for 8 h before receiving approximately 0.75 U/kg insulin via intraperitoneal injection, and blood glucose levels were measured at 0, 30, 60, and 120 min.

### Groups and treatment

Mice were divided into 5 groups: normal (continuing a normal diet), model, low-dose hucMSC-EVs, high-dose hucMSC-EVs, and metformin groups. Treatments were administered at 5, 8, 11, 14, and 17 d after STZ injection (corresponding to 33, 36, 39, 42, and 45 d after the introduction of the feeding regimen, respectively). The model and control groups received 0.1 ml of PBS via tail vein injection, while the hucMSC-EV treatment groups received tail vein injections of 20 and 30 μg of hucMSC-EVs (suspended in 0.1 ml of PBS). The metformin group was administered metformin (250 mg/kg) via oral gavage. During the treatment period, body weight and FBG levels of the mice were measured weekly. OGTT and ITT were repeated on the day before the end of the experiment. At the end of the experiment, blood was collected from the orbital sinuses of the mice into centrifuge tubes. After standing at room temperature for 1 h, the samples were centrifuged at 3,000 rpm at 4 °C for 15 min to obtain serum. Serum levels of aspartate transferase (AST; Njjcbio, Nanjing, China), alanine transaminase (ALT; Njjcbio), high-density lipoprotein cholesterol (HDL-C; Njjcbio), low-density lipoprotein cholesterol (LDL-C; Njjcbio), total cholesterol (T-CHO; Njjcbio), and TGs (Njjcbio) were measured. Pancreatic, renal, and hepatic tissues were harvested, fixed in 4 % paraformaldehyde, and embedded in paraffin for histological analysis.

### In vivo and in vitro oxidative stress detection

For the in vivo treatment, the levels of malondialdehyde (MDA; Njjcbio, Nanjing, China) and catalase (CAT; Njjcbio), as well as the activities of superoxide dismutase (SOD; Beyotime) and glutathione peroxidase (GSH-PX; Njjcbio), were determined in mouse serum according to the manufacturer’s instructions. In the in vitro treatment, hucMSC-EVs or metformin was added to the culture medium of INS-1 cells and incubated for 24 h, followed by an additional 24 h of treatment with STZ. DCF probe in ROS Assay Kit (Beyotime), live–dead cells (MeilunBio), and the levels of MDA, SOD, and CAT were then determined.

### Glucose uptake and glycogen synthesis assays

HepG2 cells were seeded at a density of 2.5 × 10^5^ cells/well in 6-well plates. After 24 h of incubation with 18 mM glucosamine hydrochloride, the medium was replaced with fresh medium containing either 10 μg/ml hucMSC-EVs, 20 μg/ml hucMSC-EVs, or 25 mM metformin. After an additional 24 h of incubation, the cells or supernatants were analyzed using the corresponding kits. An O-toluidine glucose detection kit (Beyotime) was used to measure glucose uptake in the cells. For glycogen synthesis, a glycogen content assay kit (Solarbio, Beijing, China) was used, and the glycogen content was visualized by staining with the periodic acid–Schiff staining kit (PAS; Beyotime) and observed under an Eclipse Ni-U microscope (Nikon, Tokyo, Japan).

### Apoptosis detection

INS-1 cells were seeded in 6-well plates at a density of 4 × 10^5^ cells/well. After culturing for 24 h, the medium was replaced with fresh medium containing different concentrations of hucMSC-EVs (10 or 20 μg/ml) or 25 mM metformin. After incubation for another 24 h, the medium was replaced with fresh medium containing 15.15 mM STZ, and the cells were incubated for an additional 24 h. Subsequently, the cells were collected via centrifugation at 800 rpm for 5 min and washed with PBS. The cells were stained using an apoptosis detection kit (Annexin V-FITC/PI staining; Beyotime), and cell apoptosis was analyzed using flow cytometry (Beckman).

### Determination of insulin secretion

After treating INS-1 cells with hucMSC-EVs or metformin, the cells were then incubated in Krebs–Ringer bicarbonate buffer (glucose-free) at 37 °C for 30 min. Subsequently, the cells were incubated separately in basal insulin secretion buffer (2.8 mM glucose) or glucose-stimulated insulin secretion buffer (33.3 mM glucose) at 37 °C for 1 h. After incubation, the supernatant was collected, and insulin concentration were measured using an enzyme-linked immunosorbent assay (ELISA) kit (Mkbio, Shanghai, China).

### Luciferase reporter assays

Luciferase reporter gene vectors containing either wild-type (WT) or mutated versions of the death-associated protein kinase 1 (DAPK1) binding site were constructed. These vectors were cotransfected with miR-191-5p mimic or mimic NC (Sangon, Shanghai, China) into 293T cells using a transfection reagent (UElandy, Suzhou, China). After transfection, a dual-luciferase assay kit (UElandy) was used to detect luciferase activity according to the manufacturer’s instructions.

### Gene knockdown and overexpression

INS-1 cells were transfected with miRNA NC, miRNA mimic, inhibitor NC, miRNA inhibitor, or small interfering RNA (siRNA) targeting DAPK1 (Sangon) for 48 h, followed by treatment with STZ for 24 h. Subsequent analysis included flow cytometry, protein expression assays, RNA isolation, and measurements of SOD, CAT, and MDA. Sequence information is presented in Table S1. The transfection was carried out using LipoRNAi Transfection Reagent (Beyotime).

### Western blotting

Cells and exosome lysates were homogenized in radioimmunoprecipitation assay (RIPA) buffer (Beyotime) and quantified using a BCA assay. Protein samples were subsequently separated using 10% sodium dodecyl sulfate–polyacrylamide gel electrophoresis (SDS-PAGE), followed by blocking with 5% nonfat milk in Tris-buffered saline for 2 h at room temperature. Next, the membranes were incubated overnight at 4 °C with primary antibodies, followed by incubation with horseradish peroxidase (HRP)-conjugated secondary antibodies. Chemiluminescent signals were detected using enhanced chemiluminescence (ECL) reagent (Biosharp, Anhui, China). Protein expression was normalized to β-actin levels. The primary antibodies used were as follows: TSG101 (Zenbio, Chengdu, China), CD9 (Zenbio, Chengdu, China), CD81 (Zenbio, Chengdu, China), DAPK1 (Zenbio, Chengdu, China), p-PI3K (Cell Signaling Technology, Massachusetts, USA), PI3K (Cell Signaling Technology, Massachusetts, USA), p-AKT (Cell Signaling Technology, Massachusetts, USA), AKT (Cell Signaling Technology, Massachusetts, USA), p-STAT (Cell Signaling Technology, Massachusetts, USA), STAT (Cell Signaling Technology, Massachusetts, USA), Nrf2 (ABclonal, Wuhan, China), SOD1 (ABclonal, Wuhan, China), Bcl2 (Bioss, Beijing, China), and β-actin (Yeasen, Shanghai, China). The secondary antibodies comprised HRP-conjugated goat anti-rabbit or goat anti-mouse antibodies (Bioss, Beijing, China).

### RNA isolation and qRT-PCR

Total RNA was extracted from the cells using the SPARKeasy Superpure Total RNA Kit (Sparkjade, Shandong, China) according to the manufacturer’s instructions. Reverse transcription was performed using a SPARKscript II 1st Strand cDNA Synthesis Kit (Sparkjade). Quantitative real-time polymerase chain reaction (qRT-PCR) was measured using 2× SYBR Green qPCR Mix (Sparkjade) and performed on an ABI 7500 system (Thermo Fisher Scientific, Shanghai, China). β-Actin was used as a reference control. The sequences of all specified primers are detailed in Table S2.

### Statistical analysis

Statistical analyses were performed using GraphPad Prism 10 software. All data are presented as the mean ± SD. Statistical analysis was performed using Student’s *t* test. *P < 0.05, **P < 0.01, and ***P < 0.001 were considered statistically significant.

## Results

### Extraction and identification of hucMSC-EVs

**Fig. 1.**
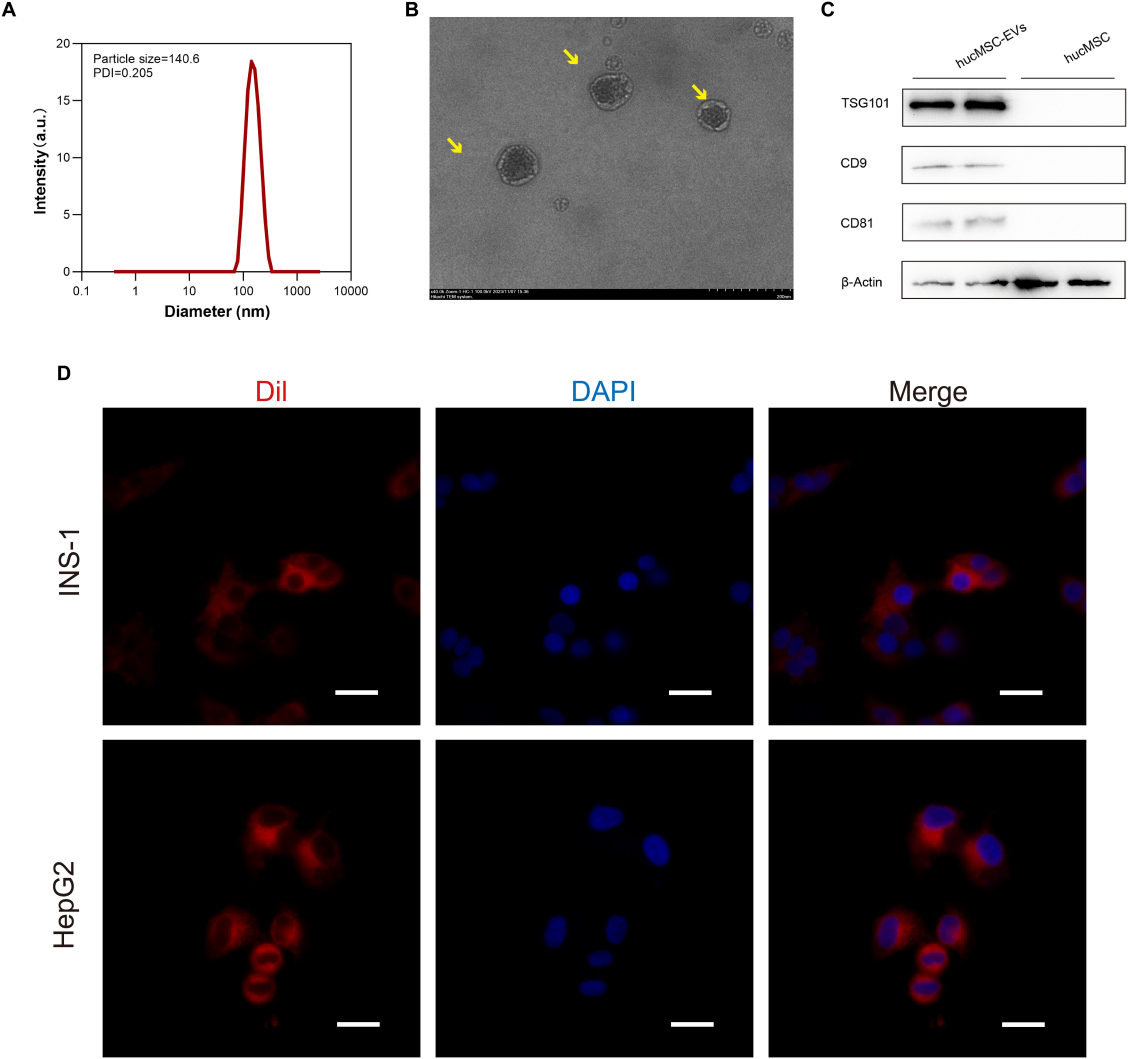
Extraction and identification of hucMSC-EVs. (A) DLS results showing the particle size distribution of hucMSC-EVs. (B) Transmission electron micrographs of hucMSC-EVs. Scale bar, 200 nm. (C) Western blotting analysis of exosomal markers TSG101, CD9, CD81, and β-actin. (D) Phagocytosis of hucMSC-EVs labeled with Dil dye by INS-1 and HepG2 cells. Scale bar, 100 μm.

hucMSC-EVs were purified from the cell culture medium using ultracentrifugation and characterized via transmission electron microscopy (TEM), dynamic light scattering (DLS), nanoflow cytometry analysis, and Western blotting. The results of TEM and DLS analysis revealed that hucMSC-EVs displayed a cup-like morphology with an average hydration particle size of approximately 140 nm (Fig. 1A and B). Additionally, nanoflow cytometry was used to evaluate extracellular vesicles size. Due to their uniform particle size and morphology, standard samples of silicon dioxide (SiO_2_) spheres of different sizes (Apogee Mix Standard) were selected as references to simulate different particle size distributions for determining the size of the hucMSC-EVs. The results of nanoflow cytometry analysis showed that the mode diameter of the hucMSC-EVs was in the range of 70 to 180 nm (Fig. S1). Furthermore, the detection of the exosomal markers TSG101, CD9, and CD81, as well as the low expression of the cytoskeletal protein β-actin, further confirmed that the isolated particles were predominantly extracellular vesicles (Fig. 1C). To identify whether hucMSC-EVs could be internalized by cells, INS-1 and HepG2 cells were treated with hucMSC-EVs stained with the red fluorescent cell membrane dye Dil. The results indicated that hucMSC-EVs could be taken up by the cells via phagocytosis (Fig. 1D).

### HucMSC-EVs alleviate IR and oxidative damage in T2DM mice

**Fig. 2.**
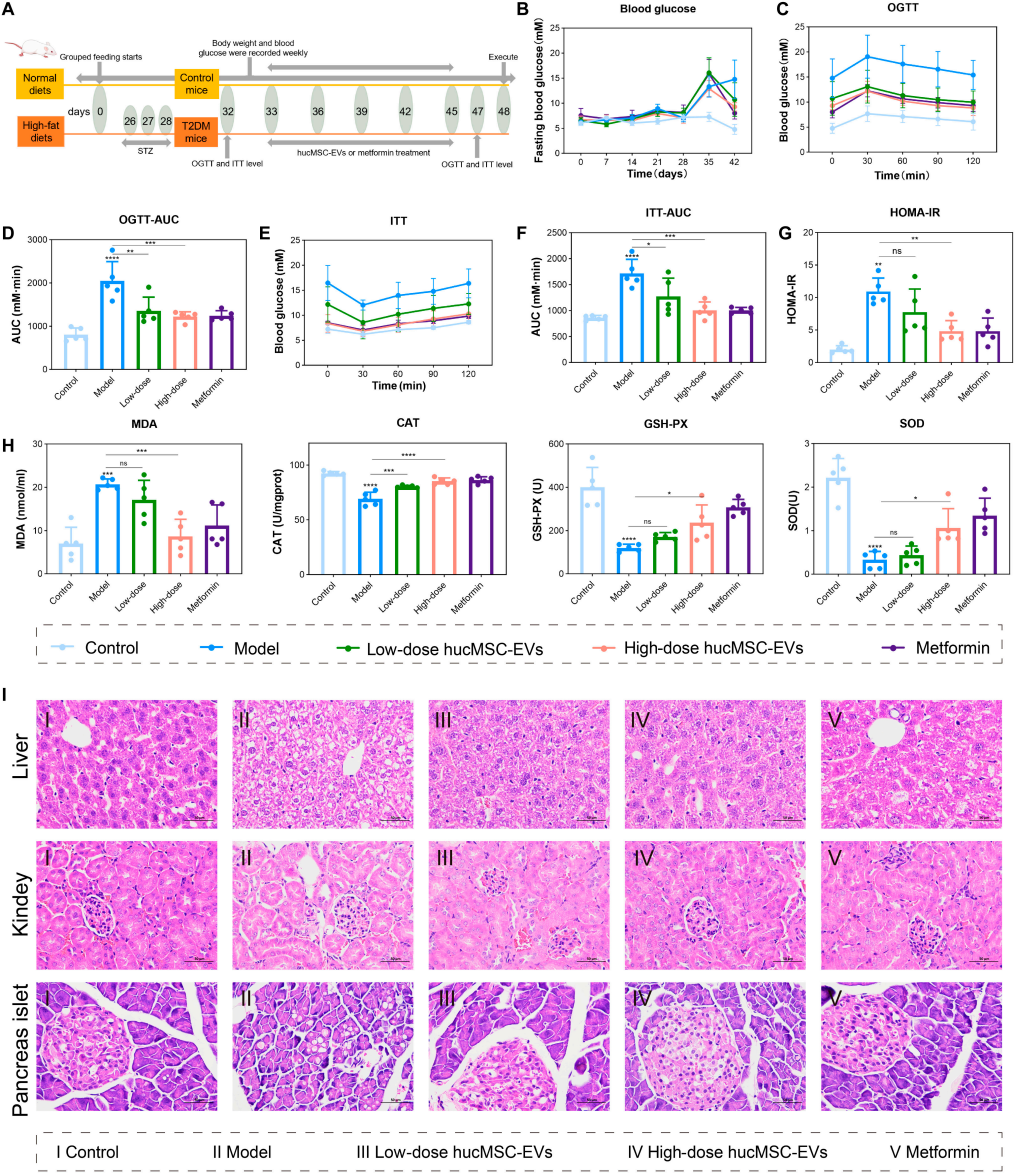
hucMSC-EVs alleviate IR and oxidative damage in T2DM mice. (A) Establishment, grouping, and administration flowchart of the animal model. (B) Weekly measurements of FBG levels. (C and D) OGTT, (E and F) ITT, and (G) HOMA-IR index recorded at 47 d after the start of the group feeding regimen. HOMA-IR = FBG value × fasting serum insulin value/22.5. (H) Serum assays showing CAT, SOD, GSH-PX, and MDA activity in all groups of mice. (I) H&E staining of mouse liver, kidneys, and pancreas. Scale bar, 50 μm. The groups contained 5 mice each; *P < 0.05, **P < 0.01, ***P < 0.001 versus Control.

To investigate the therapeutic effect of hucMSC-EVs on T2DM, we used an HFD combined with STZ to produce T2DM in BALB/c mice. Subsequently, the appropriate doses of hucMSC-EVs or the antidiabetic drug metformin were administered via tail vein injection or gavage, as shown in Fig. 2A. FBG was measured once a week, and the results showed that following intraperitoneal injection of STZ, FBG levels in the model group were notably increased compared with those in the control group (Fig. 2B). After the first round of treatment, no significant changes in FBG levels were observed among the treatment groups compared with those of the model group. However, after the third round of treatment, FBG levels in the hucMSC-EV and metformin treatment groups were notably reduced. Moreover, during the treatment process, there was no significant change in the weight of T2DM mice (Fig. S2A). On the day before the treatment endpoint, an OGTT was performed (Fig. 2C). Compared with mice in the control group, mice with T2DM showed a rapid increase in blood glucose levels to 19.8 mM 30 min after oral glucose administration, which remained high at 120 min. In contrast, at 30 min, the blood glucose levels of mice in the hucMSC-EV and metformin treatment groups were significantly lower than that in untreated T2DM mice and returned to normal levels by 120 min. A clear dose-dependent relationship was observed in both the low- and high-dose hucMSC-EV treatment groups. The area under the glucose tolerance curve provided a more intuitive confirmation of these results (Fig. 2D). As IR is the main feature that distinguishes between type 2 and type 1 diabetes, we investigated the effect of hucMSC-EVs on IR in T2DM mice using an ITT (Fig. 2E). After oral insulin administration, blood glucose levels in all groups decreased, reaching their lowest point at 30 min and returning to their initial levels at 120 min. Notably, the hucMSC-EVs and metformin-treated groups consistently showed significantly lower blood glucose levels than the model group. The area under the ITT curve and the homeostatic model assessment of IR (HOMA-IR) index provided a clearer presentation of the ability of hucMSC-EVs to significantly improve IR in a dose-dependent manner (Fig. 2F and G). These results demonstrated that hucMSC-EVs improved glucose tolerance and IR in T2DM mice.

T2DM often damages the body’s antioxidant system. Oxidative damage is closely associated with the development of T2DM and is also a trigger for IR. To explore whether hucMSC-EVs could alleviate oxidative damage in T2DM mice, we examined their effects on CAT, SOD, GSH-PX, and MDA levels in the serum of T2DM mice. The results showed that, compared with the control group, the model group exhibited significantly decreased levels of CAT, SOD, and GSH-PX and markedly increased levels of MDA, indicating a significant increase in oxidative damage in T2DM mice. In contrast, the hucMSC-EV and metformin treatment groups showed significantly elevated activities of CAT, SOD, and GSH-PX and a notable reduction in MDA content compared to the model group (Fig. 2H). Moreover, hucMSC-EVs exhibited a dose-dependent effect. These results indicated that hucMSC-EVs can effectively reduce oxidative damage in T2DM mice. In addition, the decrease in AST and ALT levels confirmed that hucMSC-EV treatment effectively alleviated STZ-induced liver damage (Fig. S2B and C). The dysregulation of glucose metabolism in T2DM leads to lipid abnormalities (decreased HDL-C, elevated LDL-C, elevated T-CHO, and elevated TGs), which significantly improved following hucMSC-EV treatment (Fig. S2D to G). These results indicate that hucMSC-EVs can effectively improve IR triggers such as oxidative damage and dyslipidemia in T2DM mice.

In addition, hematoxylin and eosin (H&E) staining revealed that the livers and kidneys of the model group mice exhibited significant steatosis, with lipid vacuoles appearing in the cytoplasm. Treatment with hucMSC-EVs significantly improved the liver cell structure, eliminated fat-containing vacuoles, and alleviated glomerular atrophy. The oxidative damage caused by high blood glucose in vivo can lead to functional damage in pancreatic β cells. Compared to the model group, hucMSC-EV treatment effectively maintained the structural integrity of pancreatic islet cells and preserved a relatively higher number of pancreatic islet cells (Fig. 2I and Fig. S2H). These studies indicate that hucMSC-EVs not only maintain the structural integrity of tissues in T2DM but also significantly improve the associated pathophysiological processes.

### HucMSC-EVs enhance insulin sensitivity and reduce β cell oxidative damage in vitro

**Fig. 3.**
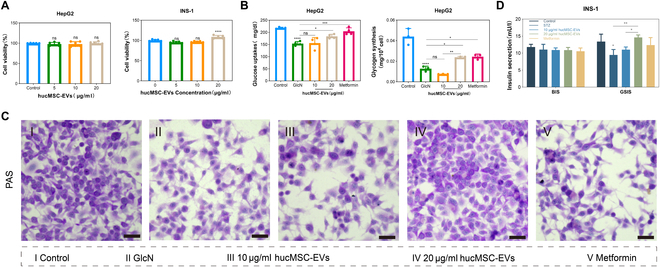
hucMSC-EVs enhanced insulin sensitivity and insulin secretion in IR-HepG2 and INS-1 cells, respectively. (A) The viability of HepG2 and INS-1 cells was assessed via Cell Counting Kit-8 (CCK8) assays after treatment with variable concentration of hucMSC-EVs for 24 h. (B and C) Effects of hucMSC-EVs on glucose uptake and glycogen synthesis, and PAS staining of glycogen following the indicated treatment in IR-HepG2 cells. Scale bar, 250 μm. (D) ELISA was conducted to measure insulin release in INS-1 cells under conditions of glucose-stimulated insulin secretion (GSIS) or basal insulin secretion (BIS). Experiments were performed in triplicate, and the results are shown as the mean ± SD. ns, no significance, *P < 0.05, **P < 0.01, ***P < 0.001, ****P < 0.0001 versus Control.

Given the functional efficacy of hucMSC-EVs in mice, we investigated their effects on hepatocytes and islet cells in vitro. First, we assessed the effect of hucMSC-EVs on the viability of HepG2 and INS-1 cells. The results showed that hucMSC-EVs had no significant influence on HepG2 cell proliferation, but it could promote INS-1 cell proliferation at a concentration of 20 μg/ml (Fig. 3A). Subsequently, we established an IR model (IR-HepG2) using HepG2 cells treated with glucosamine (Fig. S3A). In IR-HepG2 cells, hucMSC-EVs promoted glucose uptake and glycogen synthesis, as well as induced a significant concentration-dependent increase in glycogen content (Fig. 3B and C and Fig. S3C), demonstrating that hucMSC-EVs effectively improved glucose metabolism in IR-HepG2 cells. To further explore the effects of hucMSC-EVs on insulin secretion at different glucose concentrations, we established an oxidative damage model in INS-1 cells using STZ (Fig. S3B). ELISA results indicated that, compared to the control group, hucMSC-EV treatment significantly enhanced insulin secretion under high-glucose stimulation in INS-1 cells. However, under low-glucose conditions, no significant differences were observed between the groups (Fig. 3D).

**Fig. 4.**
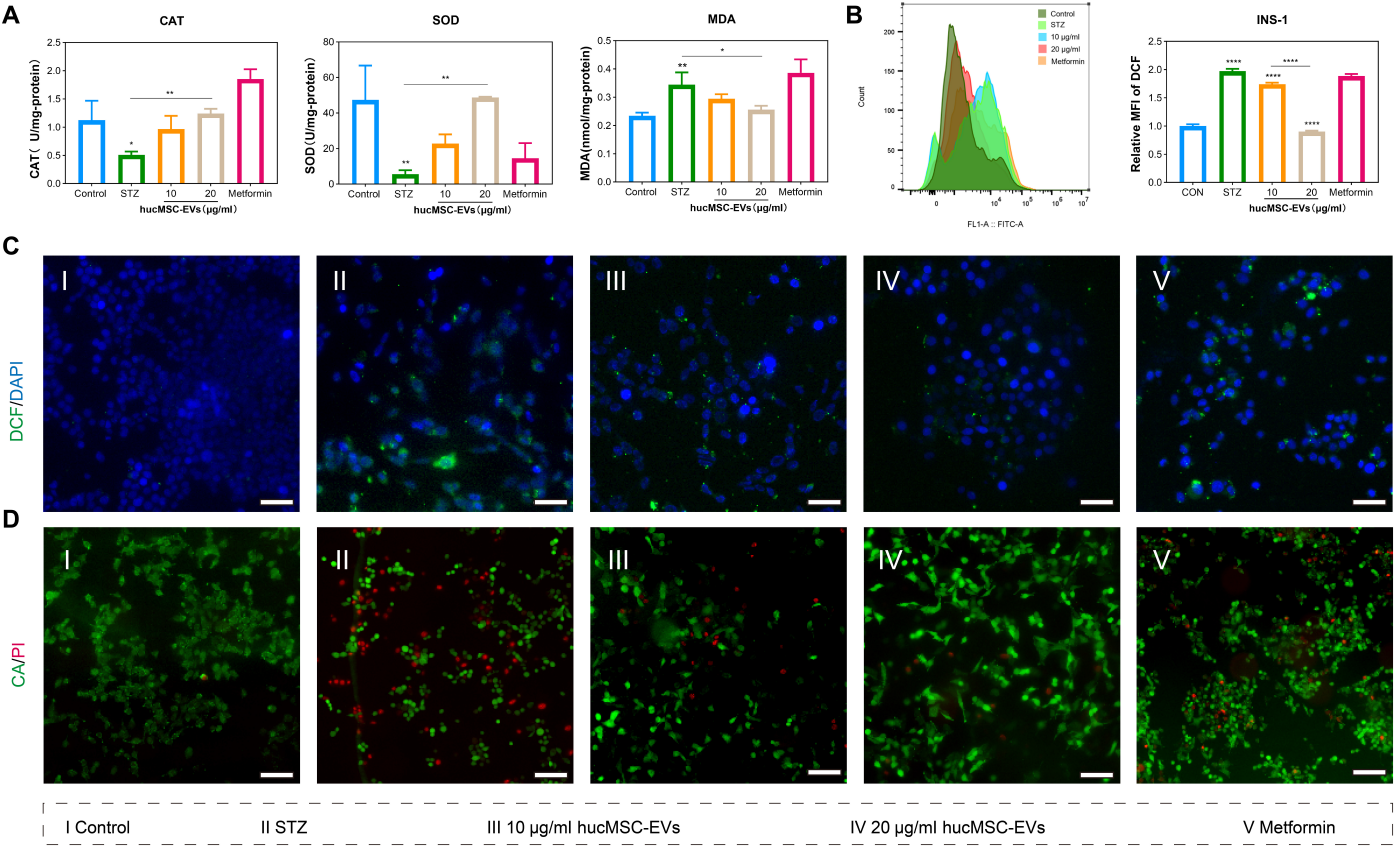
hucMSC-EVs reduced ROS generation and enhanced cell survival in the INS-1 cell oxidative damage model. (A) Measurements of MDA, CAT, and SOD activity in the INS-1 cells under the indicated treatments. (B) DCF fluorescence in the INS-1 cells was detected using flow cytometry, and the relative mean fluorescence intensity (MFI) of DCF was quantified. (C) DCF fluorescence of INS-1 cells after the indicated treatments. Scale bar, 250 μm. (D) Live–dead staining of INS-1 cells following the indicated treatments. Scale bar, 250 μm. Experiments were performed in triplicate, and the results are shown as the mean ± SD. ns, no significance, *P < 0.05, **P < 0.01, ***P < 0.001, ****P < 0.0001 versus Control.

Additionally, in the INS-1 cell oxidative damage model, compared with the STZ group, hucMSC-EV treatment led to a significant increase in the activities of CAT and SOD and a marked reduction in MDA content (Fig. 4A). To further investigate the antioxidative properties of hucMSC-EVs, we used a DCF fluorescent probe to evaluate their effect on intracellular ROS levels in INS-1 cells. These results indicated that hucMSC-EVs effectively reduced ROS levels in INS-1 cells. Compared to the STZ group, hucMSC-EV-treated cells exhibited diminished intracellular green fluorescence. Flow cytometry and fluorescence microscopy showed consistent results (Fig. 4B and C and Fig. S4). Fluorescence staining of live and dead cells also revealed a significant reduction in the number of dead cells after hucMSC-EV treatment (Fig. 4D and Fig. S5). In summary, these findings suggest that hucMSC-EVs effectively alleviate cellular IR and oxidative damage.

**Fig. 5.**
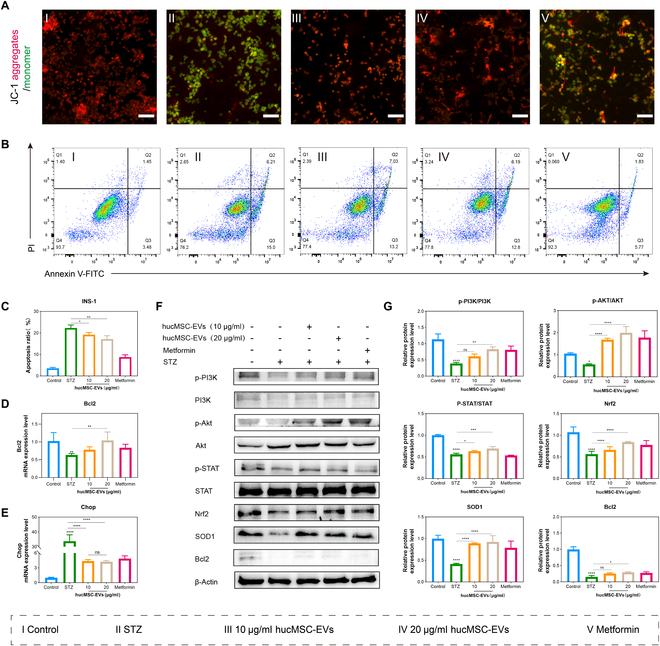
hucMSC-EVs reduce cell apoptosis, activate the PI3K/AKT and STAT signaling pathways, and increase the expression of anti-oxidative damage-related proteins. (A) Mitochondrial membrane potential (Δψ) was detected using JC-1 staining in INS-1 cells with the indicated treatments. Scale bar, 250 μm. (B and C) The level of INS-1 cell apoptosis was detected via flow cytometry, and the apoptosis ratio for each treatment group was analyzed. (D and E) Quantitative real-time PCR (qRT-PCR) analysis was performed to quantify *Bcl2* and *Chop* expression in INS-1 cells under the indicated treatments. (F and G) INS-1 cells were treated with variable concentrations of hucMSC-EVs or metformin for 24 h. Relative protein expression levels of p-PI3K, PI3K, p-AKT, AKT, p-STAT, STAT, Nrf2, SOD1, and Bcl2 were determined via Western blotting analysis. Relative quantification was conducted using ImageJ software, and the results are shown as a histogram. Experiments were performed in triplicate, and the results are shown as the mean ± SD. ns, no significance, *P < 0.05, **P < 0.01, ***P < 0.001, ****P < 0.0001 versus Control.

Normal mitochondrial membrane potential is essential for maintaining mitochondrial function and is a prerequisite for oxidative phosphorylation and ATP production. To investigate the impact of hucMSC-EVs on mitochondrial function in the oxidative damage model of INS-1 cells, we used JC-1 staining and observed a significant increase in green fluorescence, indicating a reduction in mitochondrial membrane potential. Conversely, treatment with hucMSC-EVs or metformin markedly enhanced red fluorescence, suggesting restoration of mitochondrial membrane potential (Fig. 5A and Fig. S6). Apoptosis, which is closely related to mitochondrial damage, increased following STZ treatment. However, hucMSC-EV or metformin treatment decreased the proportion of apoptotic cells (Fig. 5B and C). Furthermore, we measured the transcription levels of the apoptosis-related gene *Bcl2* and the ER stress marker *Chop*. The results showed that hucMSC-EV or metformin treatment not only restored the up-regulation of *Bcl2* but also promoted the down-regulation of *Chop* in STZ-treated cells (Fig. 5D and E). Simultaneously, mRNA and protein expression levels of *Bcl2* were increased after treatment with hucMSC-EVs (Fig. 5D, F, and G).

During STZ-induced oxidative damage in INS-1 cells, the PI3K/AKT and JAK/STAT pathways are typically inhibited, leading to decreased cell proliferation, increased apoptosis, and diminished antioxidant capacity [27]. To investigate the impact of hucMSC-EVs on the signaling pathways involved in oxidative damage, we employed Western blotting to detect the expression levels of related proteins. We found that after STZ treatment, the expression of p-PI3K (phosphorylated PI3K), p-AKT (phosphorylated AKT), and p-STAT (phosphorylated STAT) decreased, indicating that the PI3K/AKT and JAK-STAT pathways were inhibited. Conversely, after treatment with hucMSC-EVs or metformin, the expression levels of p-PI3K, p-AKT, and p-STAT significantly increased, suggesting reactivation of the PI3K/AKT and JAK/STAT pathways (Fig. 5F and G).

Nrf2 is a major antioxidant transcription factor that regulates the expression of antioxidant enzymes such as SOD1 [28]. Following STZ treatment, the expression of Nrf2 and SOD1 decreased, indicating that Nrf2 and SOD1 antioxidant capacity were inhibited. However, after treatment with hucMSC-EVs or metformin, the expression levels of Nrf2 and SOD1 increased, suggesting that their antioxidant capacities were restored (Fig. 5F and G).

### HucMSC-EV miR-191-5p mediates cellular oxidative damage and apoptosis and directly targets *DAPK1*

**Fig. 6.**
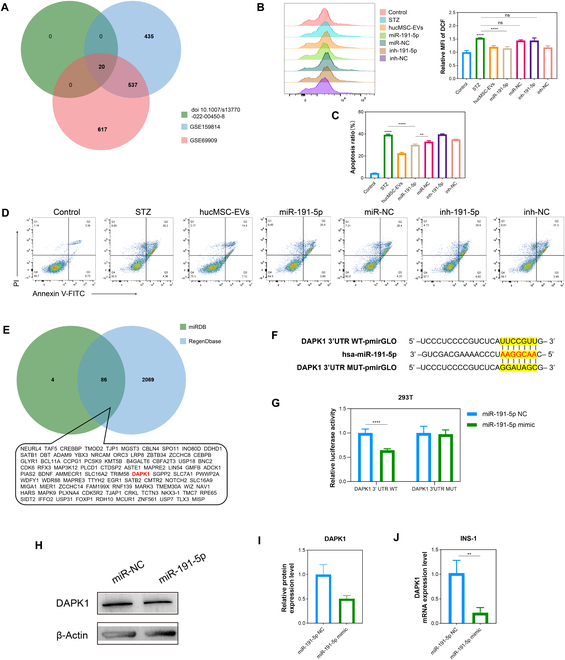
miR-191-5p mediates cellular oxidative damage and apoptosis and directly targets *DAPK1*. (A) Venn diagram of highly expressed miRNAs in hucMSC-EVs. (B) DCF fluorescence in INS-1 cells was detected via flow cytometry, and the relative MFI of DCF was statistically analyzed under the indicated treatments. (C and D) Apoptosis levels in INS-1 cells evaluated using flow cytometry, with statistical analysis of apoptosis ratios following the indicated treatments. (E) Potential targets of miR-191-5p were predicted by integrating the results from 2 databases (miRDB and RegenDbase). (F) WT and MUT binding sites between miR-191-5p and *DAPK1* are depicted. (G) Relative luciferase activity of the 293T cell line under the indicated treatments. (H and I) Western blotting analysis of DAPK1 expression was conducted for INS-1 cells under the indicated treatments. Relative quantification was performed using ImageJ software, and the results are shown as a histogram. (J) qRT-PCR assay for *DAPK1* expression in INS-1 cells under the indicated treatments. Experiments were performed in triplicate, and the results are shown as the mean ± SD. ns, no significance, *P < 0.05, **P < 0.01, ***P < 0.001, ****P < 0.0001 determined by Student’s *t* test.

We explored the mechanisms by which hucMSC-EVs attenuated oxidative damage and apoptosis. Emerging evidence indicates that numerous miRNAs are encapsulated in extracellular vesicles and play important roles in intercellular communication [25]. Therefore, we hypothesized that miRNAs within hucMSC-EVs may be involved in the regulation of oxidative stress and apoptosis in pancreatic islet cells. To validate this hypothesis, we analyzed 2 databases (GSE159814 and GSE69909) and findings from Jothimani et al. [29], identifying 20 highly abundant miRNAs in an initial screening (Fig. 6A). Sequences of the 20 miRNAs are listed in Table S3. Next, we selected 3 miRNAs that were highly conserved across generations—miR-146a-5p, miR-191-5p, and miR-146b-5p—and evaluated their effects on ROS levels and apoptosis in INS-1 cells to further elucidate their functions. The results showed that overexpression of these 3 miRNAs effectively lowered the elevated ROS levels induced by STZ, which was consistent with the results of the hucMSC-EV treatment. Notably, among the 3 miRNAs, overexpression of miR-191-5p exhibited the most significant effect in reducing ROS levels. Conversely, inhibition of the expression of these 3 miRNAs did not reduce the elevation in ROS induced by STZ (Fig. 6B and Fig. S7A and B). Additionally, the overexpression of miR-191-5p and miR-146b-5p effectively inhibited STZ-induced cell apoptosis, which was consistent with the results of the hucMSC-EV treatment. In contrast, inhibiting the expression of these 2 miRNAs did not reduce STZ-induced apoptosis (Fig. 6C and D and Fig. S7C and D). Overexpression of miR-146a-5p did not show significant differences compared to the STZ group, whereas inhibition of its expression reduced cell apoptosis, in line with previous findings (Fig. S7C and D). These results indicated that miR-191-5p may be involved in the regulation of oxidative damage and apoptosis in INS-1 cells.

miRNAs exert their biological effects by specifically binding to target mRNA recognition sites, blocking protein translation, and inducing mRNA degradation. To identify the target genes of miR-191-5p, 2 bioinformatic tools (miRDB and RegenDbase) were used to predict a set of potential target genes (Fig. 6E). Previous studies have revealed that miR-191-5p can attenuate tau phosphorylation, suppress Aβ production, and reduce neuronal cell death by regulating *DAPK1* [30]. Sequence alignment analysis of miR-191-5p and full-length *DAPK1* indicated that the 3′ untranslated region of *DAPK1* may be a potential binding site for miR-191-5p (Fig. 6I). To verify whether *DAPK1* is a target gene of miR-191-5p, we constructed both WT and mutant luciferase reporter plasmids for *DAPK1* and performed luciferase activity assays. The results showed that miR-191-5p significantly inhibited the luciferase activity of the WT *DAPK1* reporter gene, but not that of the mutant, indicating that *DAPK1* is a target gene of miR-191-5p (Fig. 6J). To further support this conclusion, we overexpressed miR-191-5p in INS-1 cells and measured the expression levels of *DAPK1*. The results revealed that the overexpression of miR-191-5p markedly reduced both the mRNA and protein expression levels of DAPK1 (Fig. 6F to H).

### MiR-191-5p/DAPK1/AKT axis alleviates oxidative damage in INS-1 cells

**Fig. 7.**
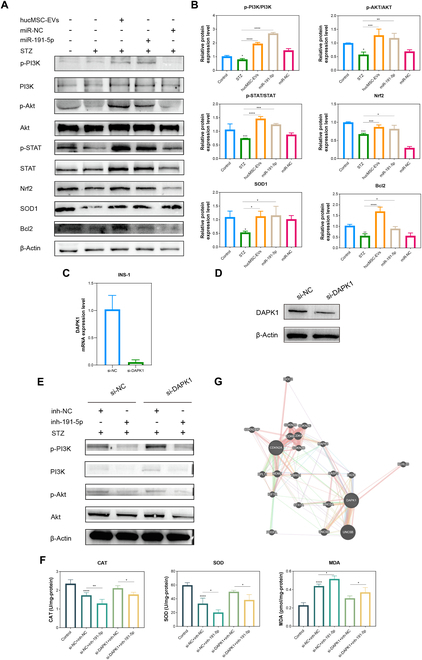
miR-191-5p alleviates oxidative damage by targeting *DAPK1* to regulate the PI3K/AKT signaling pathway. (A and B) Protein expression levels of p-PI3K, PI3K, p-AKT, AKT, p-STAT, STAT, Nrf2, SOD1, and Bcl2 were determined via Western blotting analysis in INS-1 cells under the indicated treatments. Relative quantification was conducted using ImageJ software, and the results are shown as a histogram. (C) qRT-PCR detection of *DAPK1* expression in INS-1 cells. (D) Western blotting analysis of DAPK1 expression in INS-1 cells. (E) Protein expression level of p-PI3K, PI3K, p-AKT, AKT, and β-actin was determined via Western blotting analysis following the indicated treatments. (F) Measurements of MDA content and CAT and SOD activity in INS-1 cells following the indicated treatments. (G) Protein interaction prediction map of DAPK1. Experiments were performed in triplicate, and the results are shown as the mean ± SD. Student’s *t* test was used to analyze the data. ns, no significance, *P < 0.05, **P < 0.01, ***P < 0.001, ****P < 0.0001 versus Control.

To investigate the function of miR-191-5p in oxidative damage-related signaling pathways, we overexpressed miR-191-5p and detected the expression of related signaling pathway proteins. The results showed that compared to the STZ group, the protein expression levels of p-PI3K, p-AKT, p-STAT, Nrf2, SOD1, and Bcl2 were significantly increased in samples overexpressing miR-191-5p, consistent with the results of the hucMSC-EV treatment group. In contrast, the group transfected with the control miR-NC did not show similar changes (Fig. 7A and B). Subsequently, to elucidate the role of DAPK1 in the oxidative damage-related signaling pathway, we used siRNA to knock down *DAPK1* expression in INS-1 cells and verified its knockdown efficiency via qRT-PCR and Western blotting (Fig. 7C and D). The results showed that the absence of *DAPK1* increased the expression of p-PI3K and p-AKT, revealing the regulatory role of *DAPK1* on the PI3K/AKT signaling pathway. Notably, the introduction of miR-191-5p inhibitors reversed the up-regulation of p-PI3K and p-AKT induced by *DAPK1* knockdown (Fig. 7E). These results indicate that miR-191-5p regulates the PI3K/AKT signaling pathway by targeting *DAPK1*.

Next, we knocked down *DAPK1* expression in the INS-1 cell oxidative damage model and measured the activity of the antioxidant enzymes CAT and SOD, as well as the oxidative damage marker MDA. Compared to the negative control (si-NC), *DAPK1* knockdown led to a significant increase in CAT and SOD activity and a decrease in MDA content, indicating that the absence of *DAPK1* enhanced the cellular antioxidant capacity (Fig. S8). When miR-191-5p expression was inhibited using the inhibitor inh-191-5p, CAT and SOD activity decreased, while MDA content increased compared to the si-NC + inh-NC group (Fig. 7F). These results confirmed the critical role of miR-191-5p in mitigating oxidative damage. Interestingly, the knockdown of *DAPK1* partially reversed the decrease in CAT and SOD activity and the increase in MDA content induced by inh-191-5p treatment (Fig. 7F). These results indicate that miR-191-5p affects oxidative damage by targeting *DAPK1*.

To further explore how DAPK1 contributes to T2DM regulation, protein interaction prediction tools (https://genemania.org) were used to evaluate the interactions between DAPK1 and other proteins. Among these, cyclin-dependent kinase inhibitor 2A (*CDKN2A*) was identified as a crucial regulator of the cell cycle (Fig. 7G). Recent studies have linked *CDKN2A* to T2DM progression and severity [31,32]. Therefore, we hypothesized that the miR-191-5p–DAPK1 axis alleviates oxidative damage by regulating the PI3K/AKT signaling pathway.

## Discussion

T2DM is caused by IR or pancreatic cell dysfunction. In recent years, increasing evidence has suggested that oxidative damage plays an important role in the pathogenesis of T2DM. Oxidative damage can induce or exacerbate IR [33]. ROS can interfere with insulin signaling pathways through various mechanisms, such as phosphorylation of key molecules in the insulin signaling pathway, including IRSs and PI3K, thereby inhibiting their function and reducing insulin sensitivity [34,35]. Oxidative damage can also impair pancreatic β cells, leading to β cell dysfunction, apoptosis, and reduced insulin secretion [36]. Additionally, oxidative damage can promote lipid peroxidation and disrupt lipid metabolism, resulting in elevated levels of TG and LDL-C, which further exacerbate IR and the development of T2DM [37]. Therefore, controlling and reducing ROS production and enhancing cellular antioxidant capacity are crucial for preventing and treating T2DM. Our research found that hucMSC-EVs improved cellular oxidative damage, inhibited IR, maintained liver function, improved lipid metabolism, and promoted insulin secretion, thereby effectively lowering blood glucose levels and achieving a therapeutic effect in T2DM.

The PI3K/AKT signaling pathway plays a crucial role in T2DM, contributing not only to insulin signaling and resistance but also to the regulation of oxidative damage. Insulin activates PI3K by binding to insulin receptors, which in turn activates AKT. Normal AKT signaling promotes glucose uptake and metabolism [38]. However, in T2DM, this pathway is compromised, leading to IR. PI3K/AKT signaling alleviates oxidative damage through multiple mechanisms in T2DM. First, AKT activates Nrf2 to promote its transport from the cytoplasm into the nucleus, thereby increasing the activity of antioxidant enzymes such as SOD, CAT, and GSH-PX [39–41]. Second, AKT phosphorylates Bad to prevent its binding to Bcl2, thereby maintaining the inhibitory effect of Bcl2 on apoptosis and reducing cell apoptosis. Additionally, AKT activation increases mitochondrial membrane potential, enhances mitochondrial energy generation, and elevates antioxidant capacity [42]. Moreover, the STAT pathway plays a significant role in insulin signaling, and its dysfunction may exacerbate IR [43]. Nrf2 regulates the expression of antioxidant enzymes and related proteins, such as SOD1, thereby helping cells alleviate oxidative damage, inflammation, and chronic kidney disease [41]. The endogenous anti-apoptotic protein Bcl2 interacts with Bax/Bak to prevent changes in mitochondrial membrane permeability and inhibit apoptotic signal transmission [35]. Our results indicate that hucMSC-EVs activate the PI3K/AKT and STAT signaling pathways, reducing ROS levels and apoptosis induced by oxidative damage. This effect is mediated by increased expression of Nrf2, SOD1, and Bcl2 proteins in INS-1 cells, suggesting that hucMSC-EVs regulate oxidative damage in T2DM through multiple mechanisms.

**Fig. 8.**
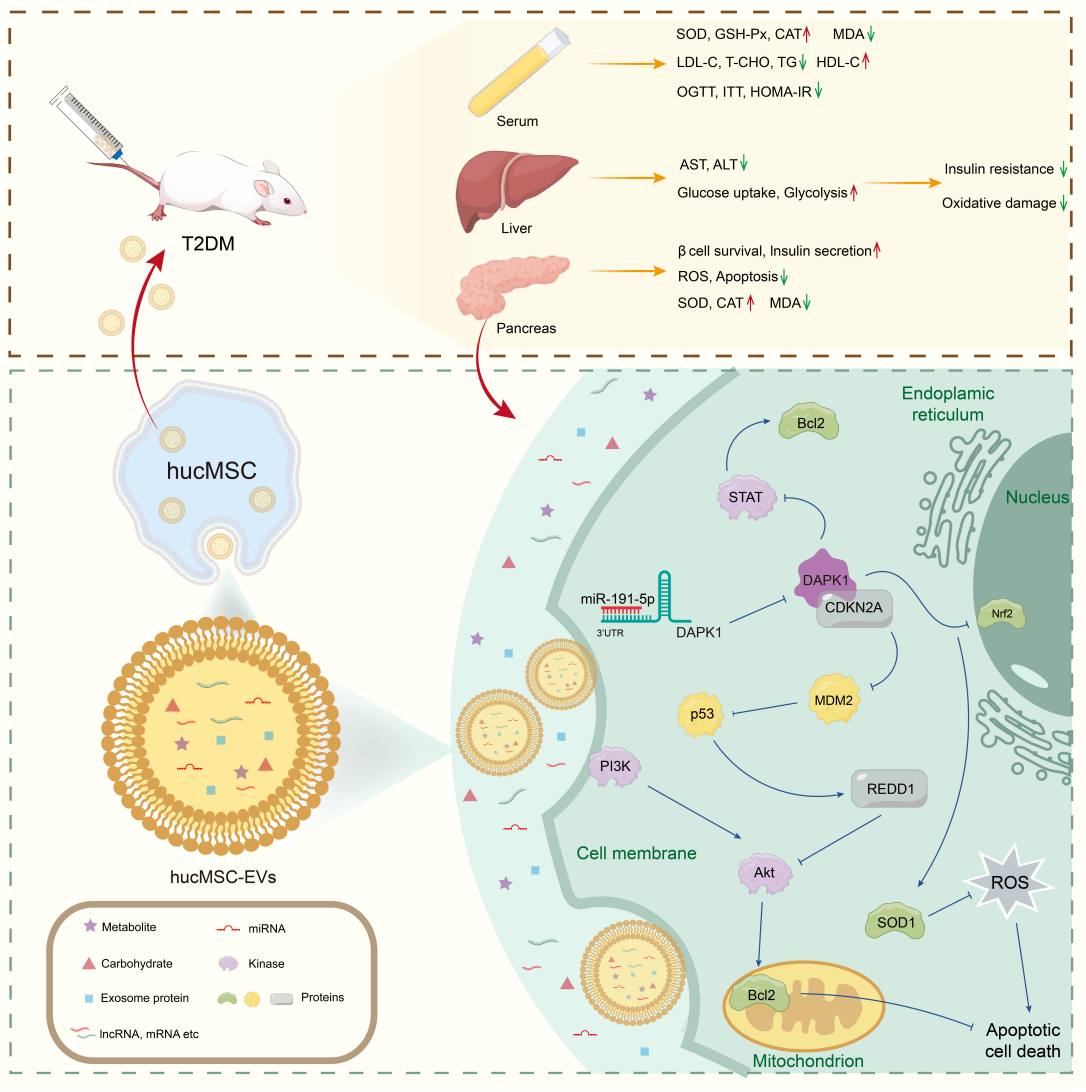
The potential mechanism of hucMSC-EVs in the regulation of oxidate damage involves the miR-191-5p/DAPK1/AKT axis in T2DM.

Extracellular vesicles can transport miRNAs from one cell to another, enabling them to affect gene expression within target cells, thereby playing roles in various biological processes, including cell communication, disease progression, and therapeutic applications. Our study highlights miR-191-5p, a highly abundant miRNA in hucMSC-EVs, which markedly reduces ROS levels and apoptosis. By targeting DAPK1, miR-191-5p enhances phosphorylation of PI3K and AKT, consequently activating the PI3K/AKT signaling pathway. *DAPK1* is a calcium-dependent serine/threonine protein kinase involved in various cellular signaling pathways, including apoptosis, autophagy, and inflammatory responses, and plays a crucial role in regulating oxidative damage. Notably, DAPK1 has also been widely implicated in treatments for tumors and neurological disorders [44]. However, its function in T2DM has rarely been investigated. Our findings indicate that hucMSC-EVs partially alleviate oxidative damage through the miR-191-5p/DAPK1/AKT axis in T2DM (Fig. 8). Our future work will involve further validating the therapeutic effects of miR-191-5p in T2DM through animal experiments.

DAPK1 is predicted to interact with CDKN2A, an essential tumor suppressor gene involved in cell cycle regulation and cellular senescence. Recent studies have indicated that *CDKN2A* expression is associated with T2DM progression and severity [31,32]. High expression of p16^INK4a^ protein encoded by *CDKN2A* may indicate severe IR and β cell dysfunction [45]. Notably, DAPK1 can indirectly reduce MDM2-mediated p53 degradation by promoting *CDKN2A* up-regulation, thereby enhancing p53 stability. The enhanced activity of p53 leads to elevated levels of REDD1, which affects the PI3K/AKT signaling pathway by inhibiting AKT phosphorylation [46]. Therefore, we hypothesized that miR-191-5p directly targets *DAPK1* and influences the PI3K/AKT signaling pathway through its regulatory effects on p53 and CDKN2A in T2DM. However, further experimental verification is necessary to explore the interaction between DAPK1 and CDKN2A. Furthermore, extracellular vesicles contain a variety of miRNAs and proteins. In future research, we plan to further explore the specific functions of these different miRNAs and to construct the interaction network of multiple miRNAs to comprehensively reveal the complex mechanism of hucMSC-EVs in the treatment of T2DM. Additionally, we will also determine the levels of miR-191-5p in serum samples from patients with T2DM. This work is expected to provide new biomarkers and potential therapeutic targets for clinical diagnosis and treatment of T2DM.

In summary, our study demonstrated that hucMSC-EVs can effectively protect pancreatic islets from damage by enhancing insulin sensitivity both in vitro and in vivo, as well as alleviate STZ-induced oxidative damage and apoptosis in INS-1 cells. Moreover, miR-191-5p, which is highly abundant in hucMSC-EVs, regulates the PI3K/AKT signaling pathway by directly targeting *DAPK1* to mitigate intracellular oxidative damage. Our findings support the potential role of hucMSC-EVs and miR-191-5p as alternative therapeutic strategies for treating T2DM.

## Data Availability

All data generated or analyzed in this study are included in the article and Supplementary Materials; further inquiries can be directed to the corresponding authors.
