# Supplementary material for: Umbilical Cord Mesenchymal Stem Cell-Derived Extracellular Vesicles Attenuate Oxidative Damage via the miR-191-5p/DAPK1/AKT Axis in Type 2 Diabetes
Source: Biomater Res. 2025 Jul 3;29:0224. doi: 10.34133/bmr.0224 (PMC12225837; doi:10.34133/bmr.0224)
Supplement: Supplementary 1 — Figs. S1 to S9 Tables S1 to S3 [file bmr.0224.f1.docx]

Supplementary Materials for

Umbilical Cord Mesenchymal Stem Cell-Derived Extracellular Vesicles Attenuate Oxidative Damage via the miR-191-5p/DAPK1/AKT Axis in Type 2 Diabetes

Anran Li ^1^, Cong Chen ^1^, Tongjia Zhang ^1^, Yuxin Tian ^1^，Yifan Cao ^1^, Xiaoming Zhao ^2, *^, and Liping Wang ^1,^ *

^1^ National Engineering Laboratory for AIDS Vaccine, Key Laboratory for Molecular Enzymology and Engineering, the Ministry of Education, School of Life Sciences, Jilin University, Changchun 130012, China

^2^ Scientific Research Center, China-Japan Union Hospital of Jilin University, Changchun 130012, China

**^*^** Address correspondence. to: Xiaoming Zhao, zhaoxiaoming@jlu.edu.cn and Liping Wang, wanglp@jlu.edu.cn, Tel.: +86-431-8515-5348

**This file includes:**

**Figures. S1 to S9**

**Tables S1 to S3**

**Supplementary Fig.S1**


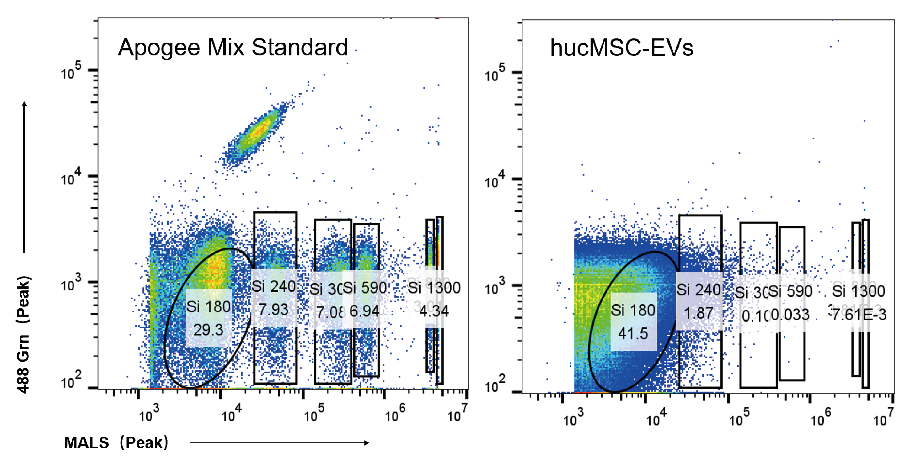


**Fig. S1 Left: Nanoflow cytometry: standard silica particle size distribution map. Right: Flow Nano Analyzer results showing the particle size distribution of hucMSC-EVs.**

**Supplementary Fig.S2**


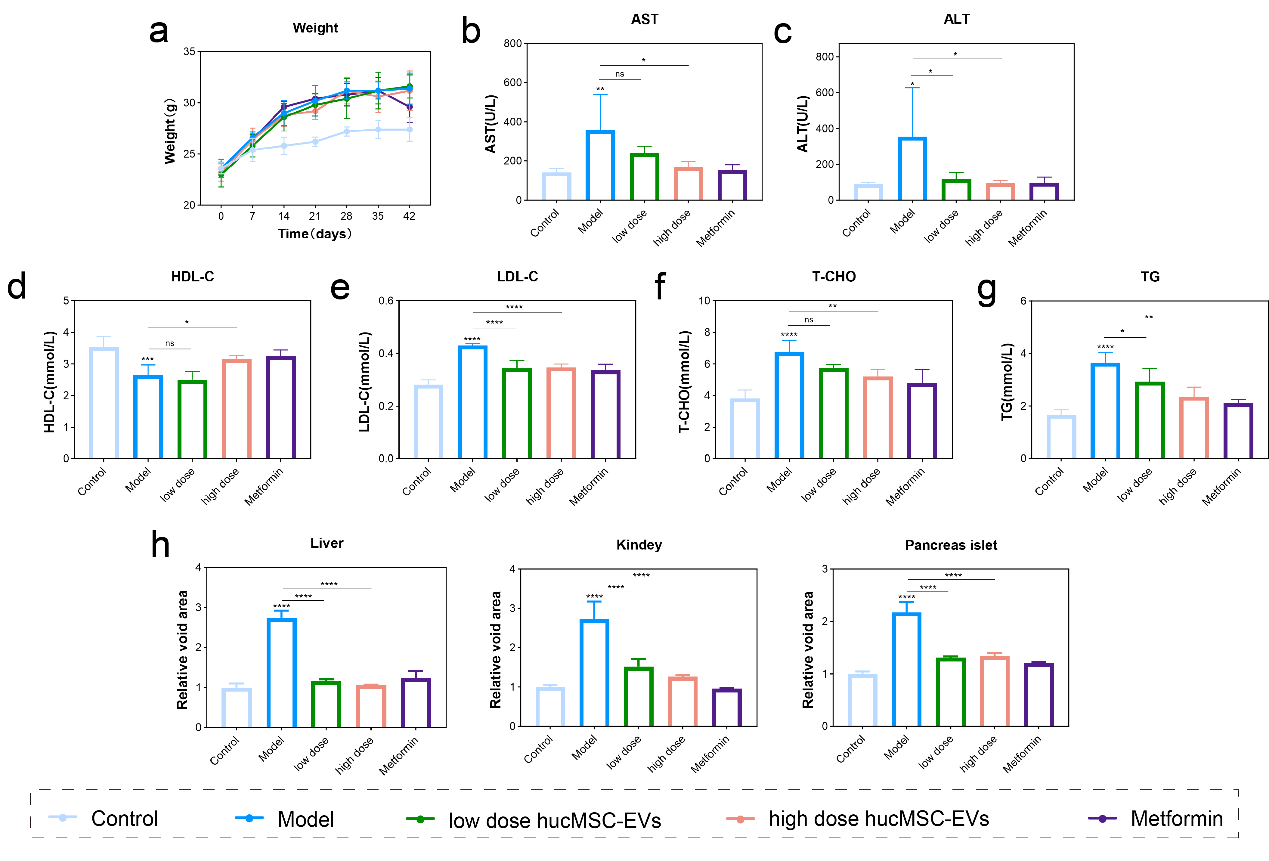


**Fig. S2** **Effects of hucMSC-EVs treatment on body weight, liver function, and blood lipid levels. (a)** Weekly body weight recordings of T2DM mice after grouped feeding. **(b–g)** Serum assays for AST, ALT, HDL-C, LDL-C, T-CHO, and TG in T2DM mice treated with varying concentrations of hucMSC-EVs or metformin. **(h)** Quantitative statistics of air bubbles in various organizations. Experiments were performed at least in triplicate, and the results are shown as the mean ± SD. ns, no significance, *p < 0.05, **p < 0.01, ***p < 0.001, ****p < 0.0001vs. Control.

**Supplementary Fig.S3**


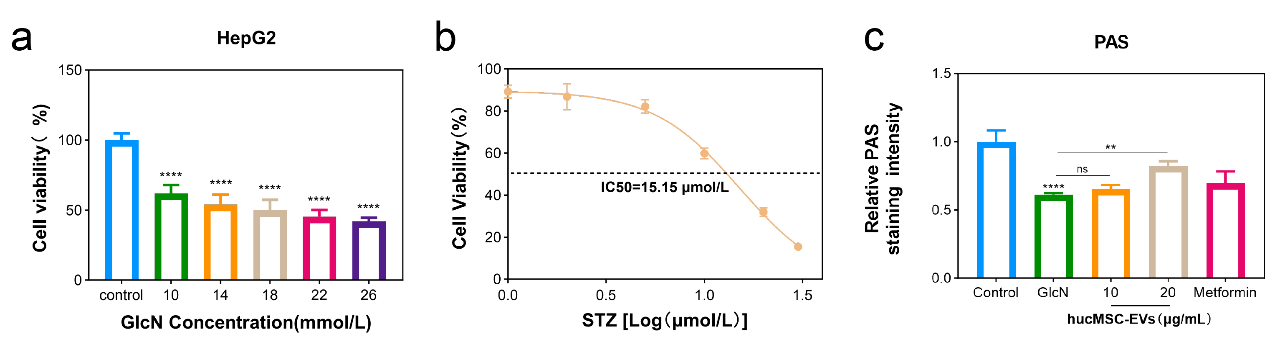


**Fig. S3 Establishment of HepG2 IR and INS-1 oxidative damage models. (a)** HepG2 cells were treated with variable concentrations of GlcN for 24 h. **(b)** INS-1 cells were treated with variable concentrations of STZ for 24 h. Cells viability was detected via CCK-8 assay, and results are shown as means ± SEM. **(c)** PAS staining quantitative analysis. Experiments were performed at least in triplicate, and the results are shown as the mean ± SD. ns, no significance, *p < 0.05, **p < 0.01, ***p < 0.001, ****p < 0.0001vs. Control.

**Supplementary Fig.S4**


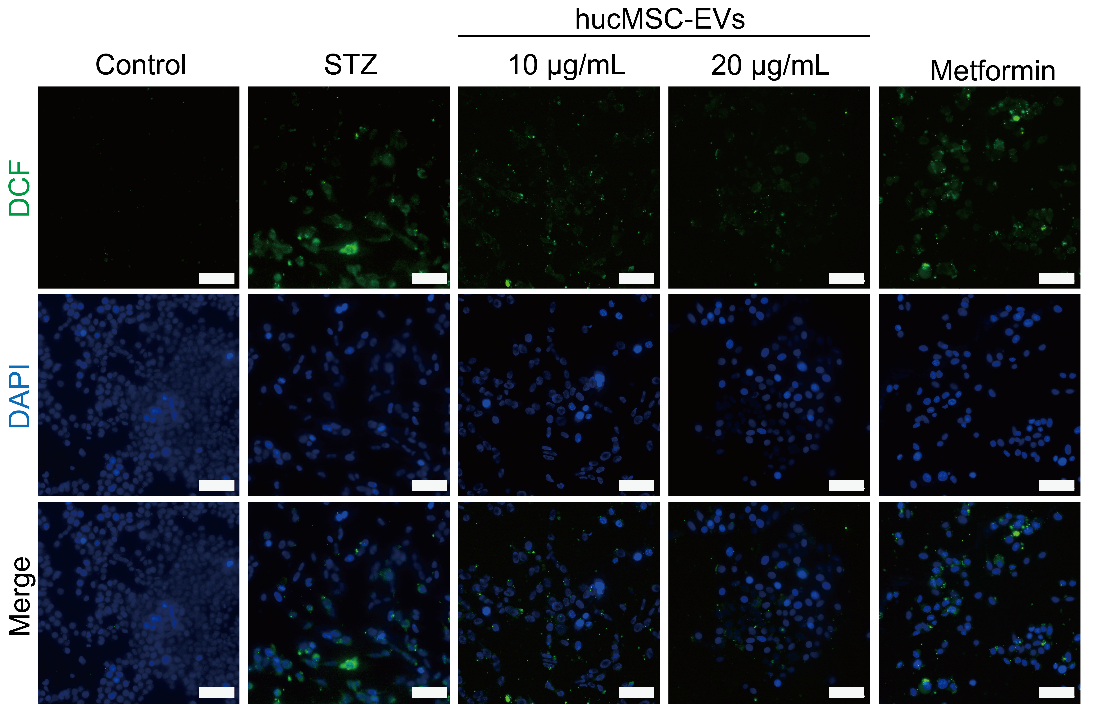


**Fig. S4 hucMSC-EVs reduce ROS levels.** DCF fluorescence of INS-1 cells after the indicated treatments. Scale bar, 250 μm.

**Supplementary Fig.S5**


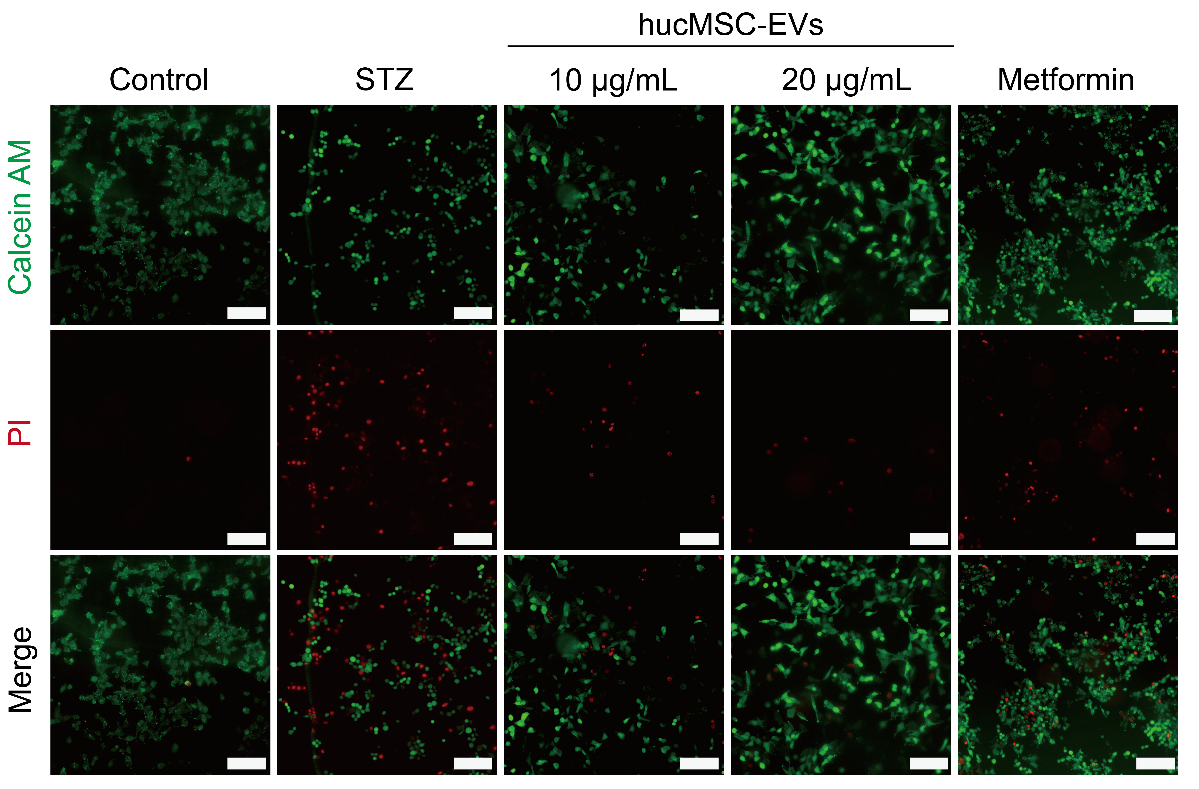


**Fig. S5 hucMSC-EVs affect live and dead cell ratios in INS-1 cells.** Live/dead staining of INS-1 cells after the indicated treatments. Scale bar, 250 μm.

**Supplementary Fig.S6**


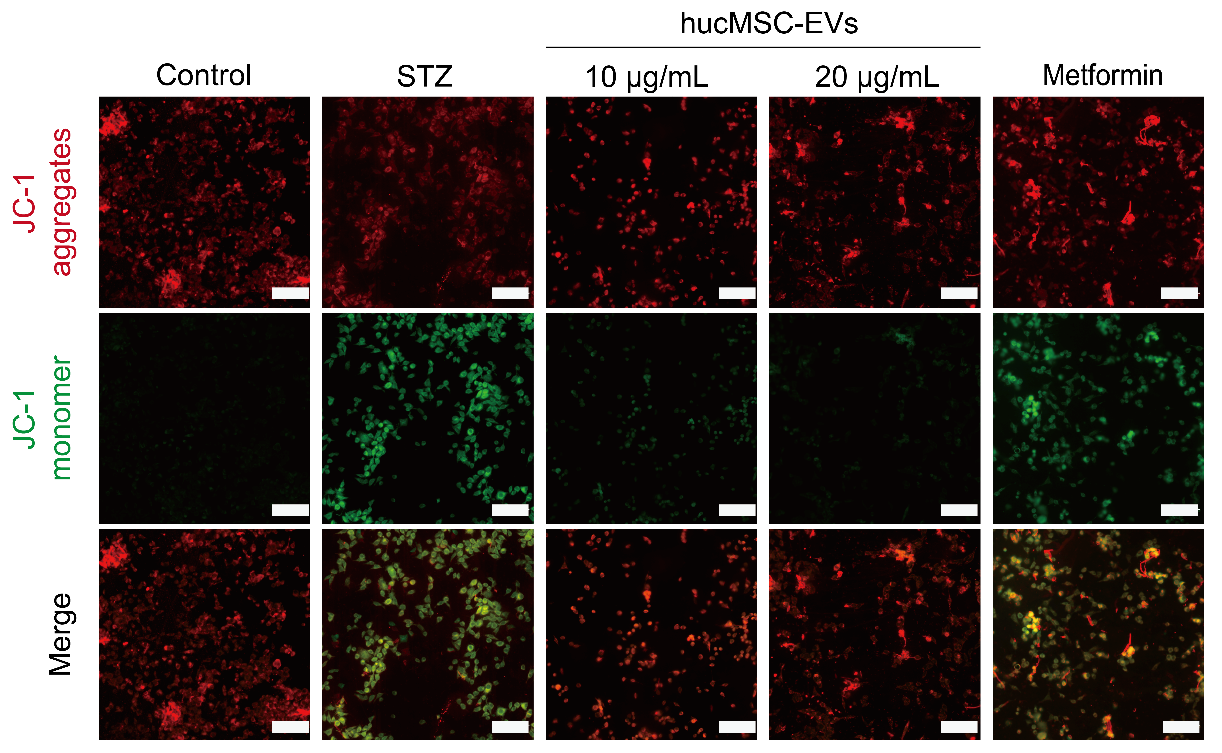


**Fig. S6 hucMSC-EVs reduce mitochondrial membrane potential.** Mitochondrial membrane potential (Δψ) was detected using JC-1 staining in INS-1 cells following the indicated treatments. Scale bar, 250 μm.

**Supplementary Fig.S7**


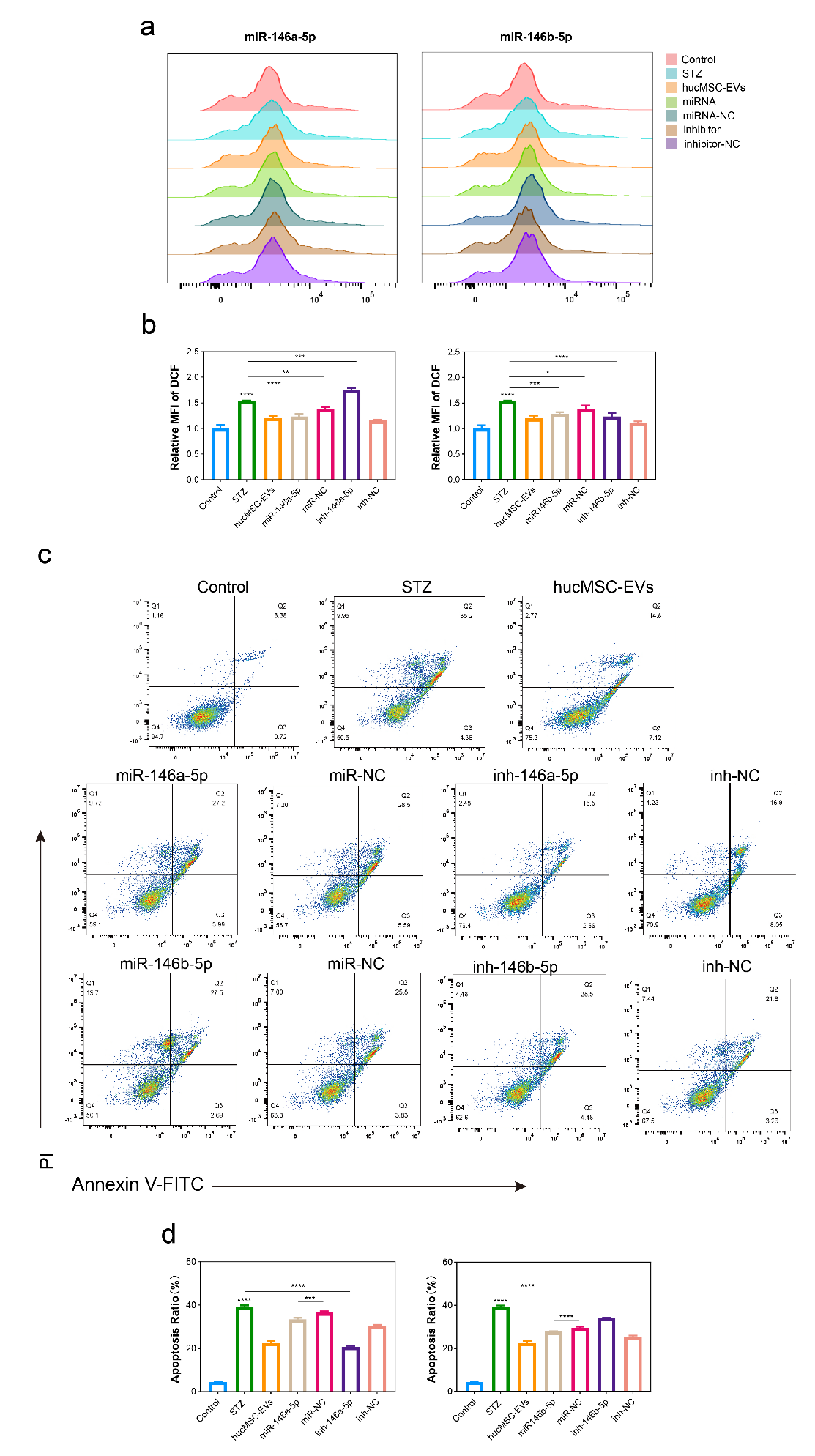


**Fig. S7 Effects of miR-146a-5p and miR-146b-5p on ROS levels and apoptosis in INS-1 cells. (a, b)** DCF fluorescence in INS-1 cells was detected via flow cytometry. Relative mean fluorescence intensity (MPI) of DCF was quantified following treatments with different miRNAs mimics and inhibitors. **(c, d)** Apoptosis levels in INS-1 cells were detected using flow cytometry, with apoptosis ratios analyzed following treatments with different miRNA mimics or inhibitors. Experiments were performed at least in triplicate, and the results are shown as the mean ± SD. ns, no significance, *p < 0.05, **p < 0.01, ***p < 0.001, ****p < 0.0001 vs. Control.

**Supplementary Fig.S8**


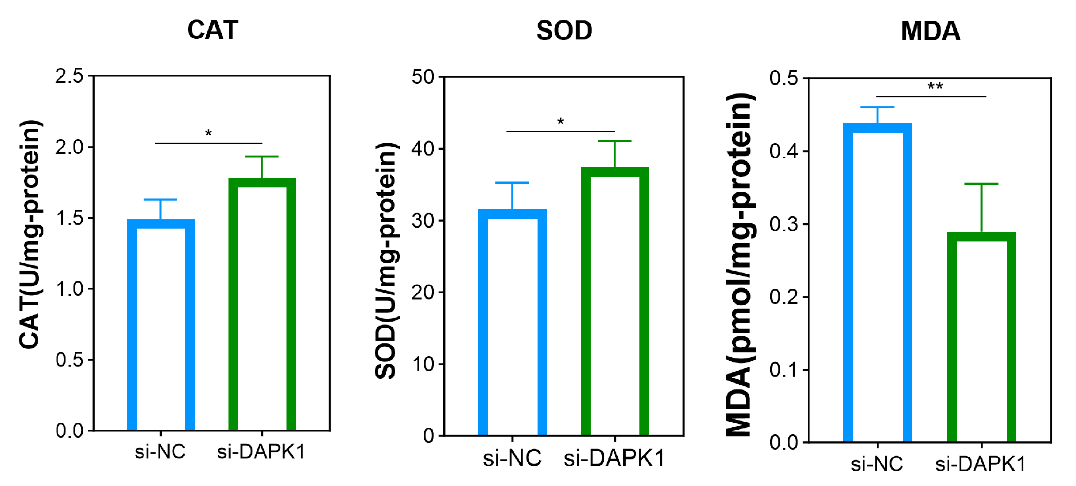


**Fig. S8 Knockdown of DAPK1 alleviates STZ-induced oxidative damage in INS-1 cells.** Measurement of MDA content and CAT and SOD activities in INS-1 cells under the indicated treatments. Experiments were performed at least in triplicate, and the results are shown as the mean ± SD. ns, no significance, *p < 0.05, **p < 0.01, ***p < 0.001, ****p < 0.0001 determined by Student′s t-test.

**Supplementary Table S1**

**Table S1. The sequences of miR-146a-5p mimic, miR-191-5p mimic, miR-146b-5p mimic, miR-146a-5p Inhibitor (inh-146a-5p), miR-191-5p Inhibitor (inh-191-5p), miR-146b-5p Inhibitor (inh-146b-5p) and siRNA targeting DAPK1.**

|  | **sense（5**′ **to 3**′**）** | **antisense（5**′ **to 3**′**）** |
| --- | --- | --- |
| miR-146a-5p mimic | UGAGAACUGAAUUCCAUGGGUU | CCCAUGGAAUUCAGUUCUCAUU |
| miR-191-5p mimic | CAACGGAAUCCCAAAAGCAGCUG | GCUGCUUUUGGGAUUCCGUUGUU |
| miR-146b-5p mimic | UGAGAACUGAAUUCCAUAGGCUG | GCCUAUGGAAUUCAGUUCUCAUU |
| inh- 146a-5p | AACCCAUGGAAUUCAGUUCUCA | — |
| inh -191-5p | CAGCUGCUUUUGGGAUUCCGUUG | — |
| inh-146b-5p | GCCTATGGAATTCAGTTCTC | — |
| si-DAPK1 | UGGGAAUAUUCAGAUGUUTT | AACAUCUGAAUAUUCCCATT |

**Supplementary Table S2**

**Table S2. The sequences of RT-qPCR primers.**

| **Primer name** | **Sequence（5**′ **to 3**′**）** |
| --- | --- |
| β-Actin-F | CCACCATGTACCCAGGCATT |
| β-Actin-R | AGGGTGTAAAACGCAGCTCA |
| Bcl2-F | GGCATCTGCACACCTGGAT |
| Bcl2-R | GCTGAGCAGCGTCTTCAGAG |
| Chop-F | GGAGTGTACCCAGCACCATC |
| Chop-R | CAAGCCCCTCTCCTTTGGTC |
| DAPK1-F | GAGGCAGACATGTGGAGCAT |
| DAPK1-R | ATCCTTGACCAGCAGCCTTC |

**Supplementary Table S3**

**Table S3. High abundance miRNAs in hucMSC-EVs.**

| **miRNA** | **Sequence（5**′ **to 3**′**）** |
| --- | --- |
| hsa-miR-21-5p | UAGCUUAUCAGACUGAUGUUGA |
| hsa-miR-29a-3p | UAGCACCAUCUGAAAUCGGUUA |
| hsa-miR-146a-5p | UGAGAACUGAAUUCCAUGGGUU |
| hsa-miR-125b-5p | UCCCUGAGACCCUAACUUGUGA |
| hsa-miR-222-3p | AGCUACAUCUGGCUACUGGGU |
| hsa-miR-27a-3p | UUCACAGUGGCUAAGUUCCGC |
| hsa-miR-100-5p | AACCCGUAGAUCCGAACUUGUG |
| hsa-miR-125a-5p | UCCCUGAGACCCUUUAACCUGUGA |
| hsa-miR-27b-3p | UUCACAGUGGCUAAGUUCUGC |
| hsa-let-7i-5p | UGAGGUAGUAGUUUGUGCUGUU |
| hsa-miR-92a-3p | UAUUGCACUUGUCCCGGCCUGU |
| hsa-miR-191-5p | CAACGGAAUCCCAAAAGCAGCUG |
| hsa-miR-16-5p | UAGCAGCACGUAAAUAUUGGCG |
| hsa-miR-146b-5p | UGAGAACUGAAUUCCAUAGGCUG |
| hsa-miR-148a-3p | UCAGUGCACUACAGAACUUUGU |
| hsa-let-7g-5p | UGAGGUAGUAGUUUGUACAGUU |
| hsa-miR-25-3p | CAUUGCACUUGUCUCGGUCUGA |
| hsa-miR-20a-5p | UAAAGUGCUUAUAGUGCAGGUAG |
| hsa-miR-15b-5p | UAGCAGCACAUCAUGGUUUACA |
| hsa-miR-23b-3p | AUCACAUUGCCAGGGAUUACCAC |
